# Effects of a Digital Therapeutic Adjunct to Eating Disorder Treatment on Health Care Service Utilization and Clinical Outcomes: Retrospective Observational Study Using Electronic Health Records

**DOI:** 10.2196/59145

**Published:** 2024-11-27

**Authors:** Jorge E Palacios, Kathryn K Erickson-Ridout, Jane Paik Kim, Stuart Buttlaire, Samuel Ridout, Stuart Argue, Jenna Tregarthen

**Affiliations:** 1 Bright Therapeutics San Francisco, CA United States; 2 Kaiser Permanente Oakland, CA United States; 3 Stanford University Stanford, CA United States

**Keywords:** digital therapeutics, app-augmented therapy, eating disorders, health care utilization, costs, real-world data, depression, emergency department, outpatient care, eating, treatment, therapy, retrospective analysis, electronic health record, patient, app, outpatient

## Abstract

**Background:**

The need for scalable solutions facilitating access to eating disorder (ED) treatment services that are efficient, effective, and inclusive is a major public health priority. Remote access to synchronous and asynchronous support delivered via health apps has shown promise, but results are so far mixed, and there are limited data on whether apps can enhance health care utilization.

**Objective:**

This study aims to examine the effects of app-augmented treatment on clinical outcomes and health care utilization for patients receiving treatment for an ED in outpatient and intensive outpatient levels of care.

**Methods:**

Recovery Record was implemented in outpatient and intensive outpatient services in a California-based health maintenance organization. We examined outcomes for eligible patients with ED by comparing clinical and service utilization medical record data over a 6-month period after implementation with analogous data for the control group in the year prior. We used a logistic regression model and inverse-weighted estimates of the probability of treatment to adjust for treatment selection bias.

**Results:**

App-augmented treatment was associated with a significant decrease in emergency department visits (*P*<.001) and a significant increase in outpatient treatment utilization (*P*<.001). There was a significantly larger weight gain for patients in low-weight categories (ie, underweight, those with anorexia, or those with severe anorexia) with app-augmented treatment (treatment effect: 0.74, 0.25, and 0.35, respectively; *P*=.02), with a greater percentage of patients moving into a higher BMI class (*P*=.01).

**Conclusions:**

Integrating remote patient engagement apps into ED treatment plans can have beneficial effects on both clinical outcomes and service utilization. More research should be undertaken on long-term efficacy and cost-effectiveness to further explore the impact of digital health interventions in ED care.

## Introduction

The need for scalable solutions facilitating access to eating disorder (ED) treatment services that are efficient, effective, and inclusive is a major public health priority. EDs continue to be a large burden on populations across the globe, with recent reviews suggesting a lifetime prevalence of any ED of 1.69% [1]. The global burden of disease attributable to ED has risen sharply in recent years, and factors including emerging food technology and weight stigma exacerbated through social media [2] could perpetuate the trend toward increasing ED prevalence over the coming years.

Despite some progress being made in addressing treatment access barriers, most individuals with ED either fail to seek or fail to adequately participate in specialized care. Reviews have found that more than half of identified individuals with an ED have never accessed treatment for their condition [3]. For those who do access specialized care, dropout rates are high, with up to 73% of patients with ED dropping out of outpatient treatment [4]. A contribution to high dropout may be barriers related to the dissemination and implementation of empirically supported treatments for ED, with clinicians reporting concerns about the generalizability of research findings and modifying existing treatment in clinical practice [5]. Without adequately participating in evidence-based treatment, individuals may experience a longer duration of illness, worsening of clinical outcomes, and higher rates of hospitalization and rehospitalization [6,7]. In the United States, health care utilization data show that inpatient admissions for EDs doubled in 2019, and the length of stay also increased from a median of 9 days between June and December 2019 to a median of 12 days over the same period in 2020 [8]. Such high utilization contributes to the economic costs of EDs in the United States, estimated recently to be US $64.7 billion (95% CI US $63.5 to US$66.0 billion) [9]. Furthermore, 20 studies were included in a systematic review on the economic burden of anorexia nervosa (AN), bulimia nervosa (BN), and binge eating disorder (BED), finding an association for all 3 conditions with increased health service use, which included emergency, inpatient, and outpatient care [10].

Contemporary digital therapeutic (DTx) adjuncts such as cognitive behavioral therapy (CBT) apps and remote patient engagement systems are innovations that may support the effective implementation of, and patient engagement in, evidence-based treatments for ED [11-13]. Specifically, for overburdened practitioners with large caseloads, these tools may facilitate higher “doses” of synchronous and asynchronous support through app-delivered, day-to-day interventions and brief, therapist-delivered feedback [14]. Transitioning away from burdensome, paper-based, cognitive-behavioral meal monitoring and journaling toward a responsive digital format may increase completion rates and thus better support practitioners in the delivery of personalized care, guided by data on relevant areas of clinical concern [15]. Finally, such tools may facilitate timely help seeking by removing geographical barriers, thereby preventing escalation of symptoms and the need for higher levels of care [16].

Such DTx adjuncts for ED have demonstrated promising early outcomes in terms of their effectiveness [17,18]. However, research examining the real-world applicability of these digital innovations, app-based interventions, and their impact on health care service utilization remains scarce. One systematic review on cost-effectiveness studies for general, nondigital interventions for ED first published in 2017 identified 13 studies and had inconclusive findings, with interventions being either cost-saving or effective and more costly than their comparators [19]. More recently, a randomized controlled trial (RCT) incorporating a web-based, unguided self-help program was conducted with an accompanying cost-effectiveness study. The authors found nonsignificant differences in favor of the web-based program with regard to costs and quality of life–adjusted life-years between all 4 conditions, which included the web-based unguided program, expert-patient support via email, the program and support combined, and treatment as usual (TAU) [20]. However, to our knowledge, no study has yet focused on the impact of health care utilization upon the implementation of app-augmented treatment (AAT) for ED.

Consequently, the focus of this study is to assess the effects of incorporating an AAT that offers a remote patient engagement system in outpatient and intensive outpatient ED treatment settings. The AAT includes TAU plus a CBT-based mobile app that delivers therapeutic self-monitoring tasks and just-in-time therapeutic interventions to patients multiple times per day, which are tied to diagnosis-specific treatment goals. Therefore, we examined how receiving AAT compared with TAU with regard to health outcomes as well as ambulatory and acute psychiatric service utilization within a large, integrated health maintenance organization (HMO).

## Methods

### Setting

This is a retrospective observational study emulating a pragmatic, clustered RCT (see Table 1 for comparison of the specified and emulated target trial design features) via the reporting and analysis of data collected within the medical record system of Kaiser Permanente Northern California (KPNC) and the Recovery Record (RR) mobile ED management program app.

KPNC is a large, integrated health care system serving more than 4.6 million patients (36% of the regional population) insured through commercial, Medicare, Medicaid, and health insurance exchange plans. KPNC patients are highly representative of the ethnic and socioeconomic diversity of the surrounding and statewide population [21].

**Table 1:** Target trial protocol (specification and emulation).

Protocol component	Description	Randomized controlled trial specification	Emulation in this study using observational cohorts
Eligibility criteria	Who will be included in the study?	Patients with an ICD^a^ diagnosis for an eating disorder; age ≥13 years	Same as for specification. Required data for each person: primary diagnosis, age, treatment appointment status, and treatment strategy assignment.
Treatment strategies	What interventions will eligible persons receive?	App-augmented treatment: treatment as usual + Recovery Record app Treatment as usual: psychotherapy, dietetic support, and medication management.	Same as for specification. Required data: baseline appointment with 1 of the 2 interventions.
Treatment assignment	How will eligible persons be assigned to the interventions?	Pragmatic trial without blind assignment. Participants are randomly assigned to either strategy and are aware of the strategy to which they have been assigned.	Eligible persons will be assigned to the strategies with which their data were compatible at the time of eligibility. Inverse probability score weighing performed to emulate the random assignment of treatment strategies.
Outcome	What outcomes in eligible persons will be compared among intervention groups?	Health outcomes: change in BMI, blood pressure, and depressive symptoms at the end of treatment compared with baseline. Health care utilization: outpatient compliance and retention (psychiatry and primary care) and emergency department utilization during treatment.	Same as for specification. Required data: baseline and follow-up BMI, blood pressure, depressive symptoms, outpatient psychiatry and primary care visits, and emergency department visits
Follow-up period	During which period will eligible persons be followed in the study?	Starts at baseline and ends after baseline	Same as for specification. Required data: date of loss to follow-up
Causal estimated	Which counterfactual contrasts will be estimated using the above data?	Complete case analysis (effect of receiving treatment)	Inverse probability treatment weighted average treatment effect of the AAT^b^
Statistical analysis	How will the counterfactual contrasts be estimated?	Effect estimated via comparison of change in health outcomes from baseline among individuals assigned to each treatment strategy.	Same as complete case analysis inverse probability treatment weighting

^a^ICD: International Classification of Diseases.

^b^AAT: app-augmented treatment.

While individuals who are members of the majority of health plans in the United States typically receive treatment from multiple providers using different medical records, KPNC members receive all health care services exclusively with KPNC providers who use a singular medical record (Epic Systems). This created the unique opportunity to access a complete data set on clinical and health care service utilization outcomes.

### Ethical Considerations

All patients using RR consent to the anonymized use of their data for research and quality improvement when they access the software, as per RR’s privacy policy [22]. In addition, the software is fully Health Insurance Portability and Accountability Act compliant. As this paper presents an evaluation of an ongoing health service, no approval of a research ethics committee was obtained. Since the study was classified as a quality improvement study and deemed to be of minimal risk, no approval of a research ethics committee was deemed necessary by KPNC. All data included were deidentified prior to analysis. The research was conducted in accordance with the principles of the Declaration of Helsinki. No compensation was provided to participants.

### Study Population

All included patients received treatment for an ED at 1 of 6 participating KPNC ED treatment programs and were divided into the TAU or ATT group; those in the AAT group were patients who had also successfully linked with a member of their treatment team using the RR app. All patients had received a diagnosis for an ED, per *International Classification of Diseases, Tenth Revision* (*ICD-10*) codes in the medical record, and were continuing members of KPNC, to ensure that they met inclusion criteria of having had access to KPNC’s services and complete medical record data for the full run-period of the study (6 months). Members whose health insurance with KPNC was discontinued during the study period were excluded (n=21). Of those continuing members who were identified, we excluded those who were younger than 13 years (n=4); had incomplete data at baseline (n=2666); and those with an *ICD-10* code of F98.X, relating to “other behavioral and emotional disorders with onset usually occurring in childhood and adolescence” (n=738). We also excluded those who had a BMI outside the plausible range, that is, less than 8 kg/m^2^ or above 60 kg/m^2^ (n=33).

### Interventions

#### TAU Group

Patients with ED treated in the department of psychiatry received a combination of psychotherapy, dietetic support, and medication management where relevant with a mental health therapist, dietitian, and psychiatric prescriber. Treatment was scheduled at the discretion of the therapist and the patient at regular intervals and could have included a combination of groups, individual therapy, and medication management when indicated. Other specifics of treatment followed American Psychological Association guideline–recommended components including assessment of weight, eating behaviors, weight control behaviors, prior treatment, goal setting, and caregiver engagement [23].

#### ATT Group

The AAT is a hybrid intervention that combines face-to-face and digital care. Patients received in-person TAU plus linkage to their primary therapist’s RR clinician app, registered dietitian’s RR clinician app, or both. This connection served to foster the DTx relationship and associated accountability, and to extend this relationship into patients’ daily lives. Face-to-face time was supplemented with in-the-moment digital check-ins; patient receipt of in-app feedback from their clinical team; and collaborative, regular review of clinical goals and therapeutic outcomes, which were collected via a standardized monthly questionnaire. Patients using RR as a part of their treatment also had the option to continue using the tool after discharge free of charge to help maintain progress.

**Figure 1:**
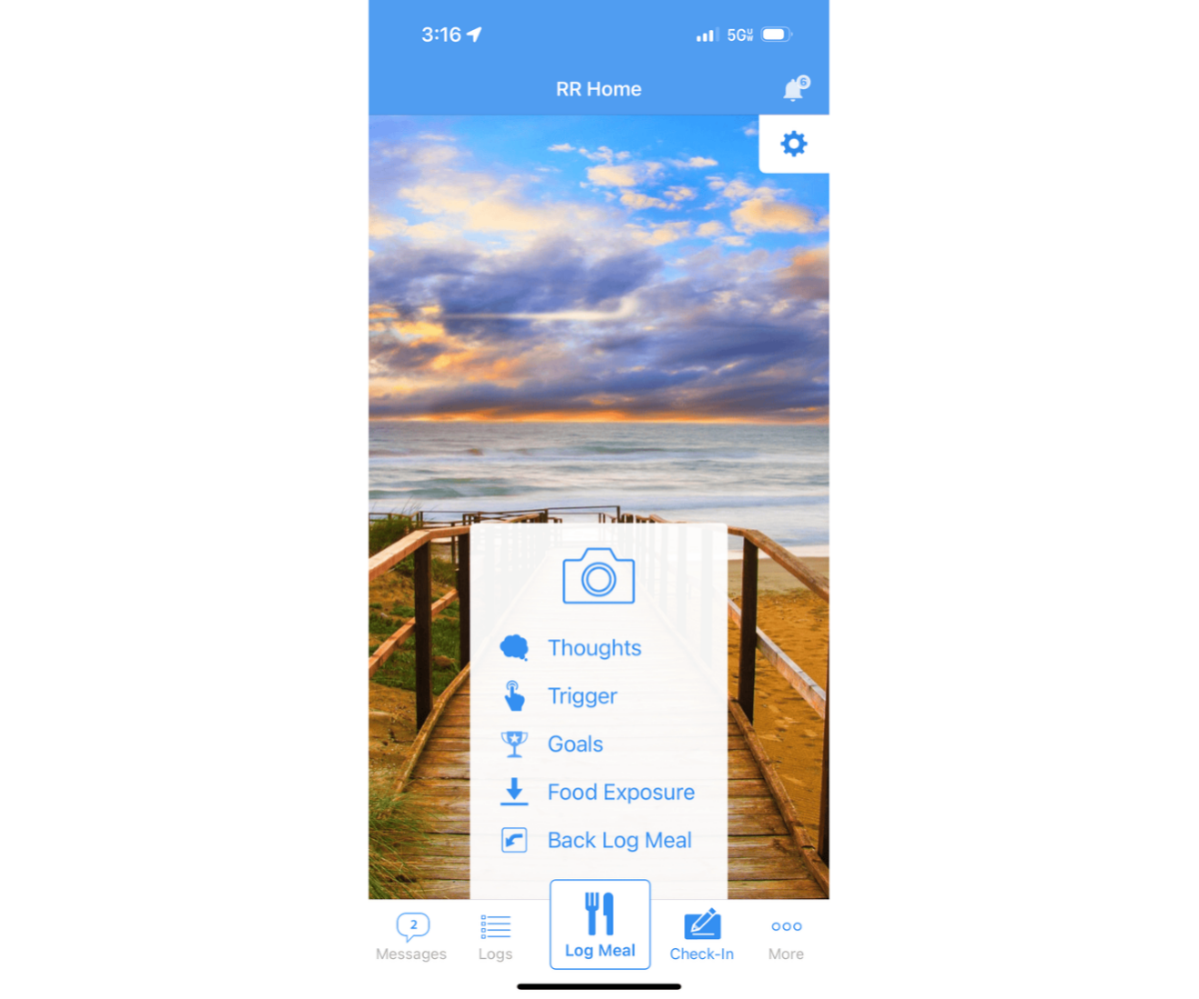
Home screen of the Recovery Record (RR) app.

**Figure 2:**
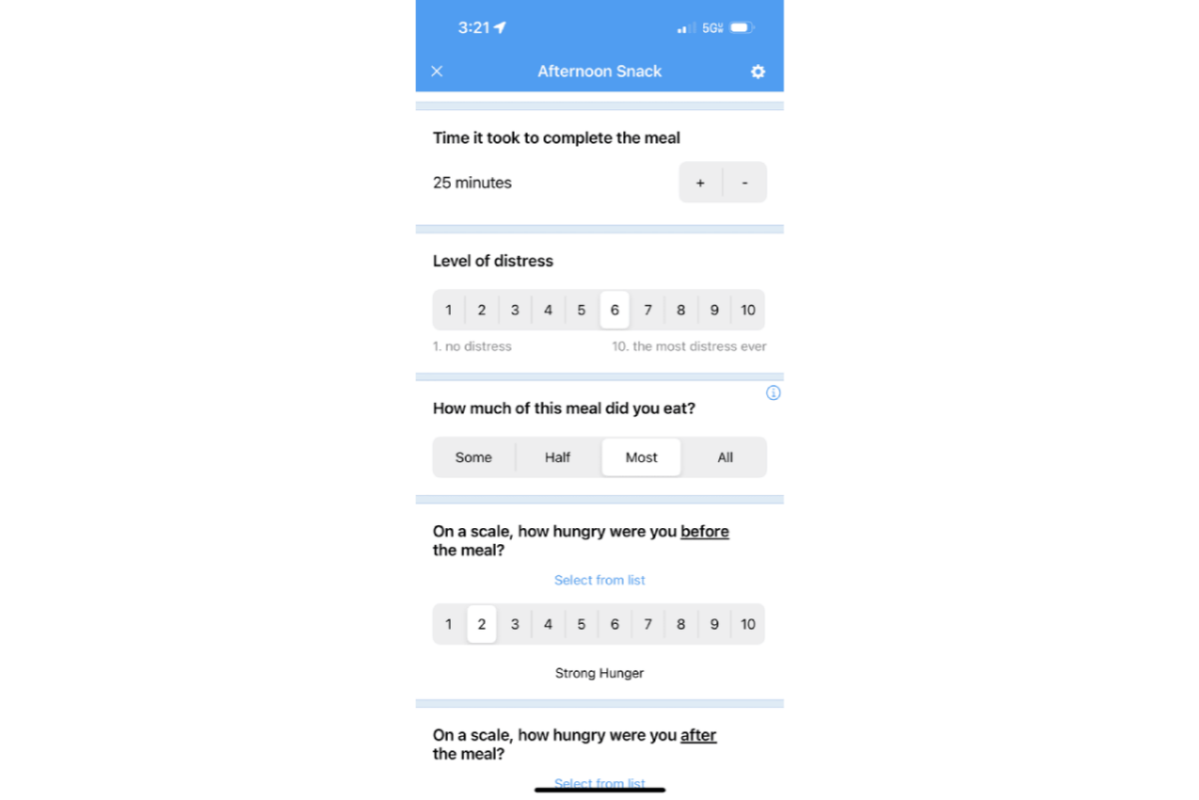
Example meal log on the Recovery Record app.

**Figure 3:**
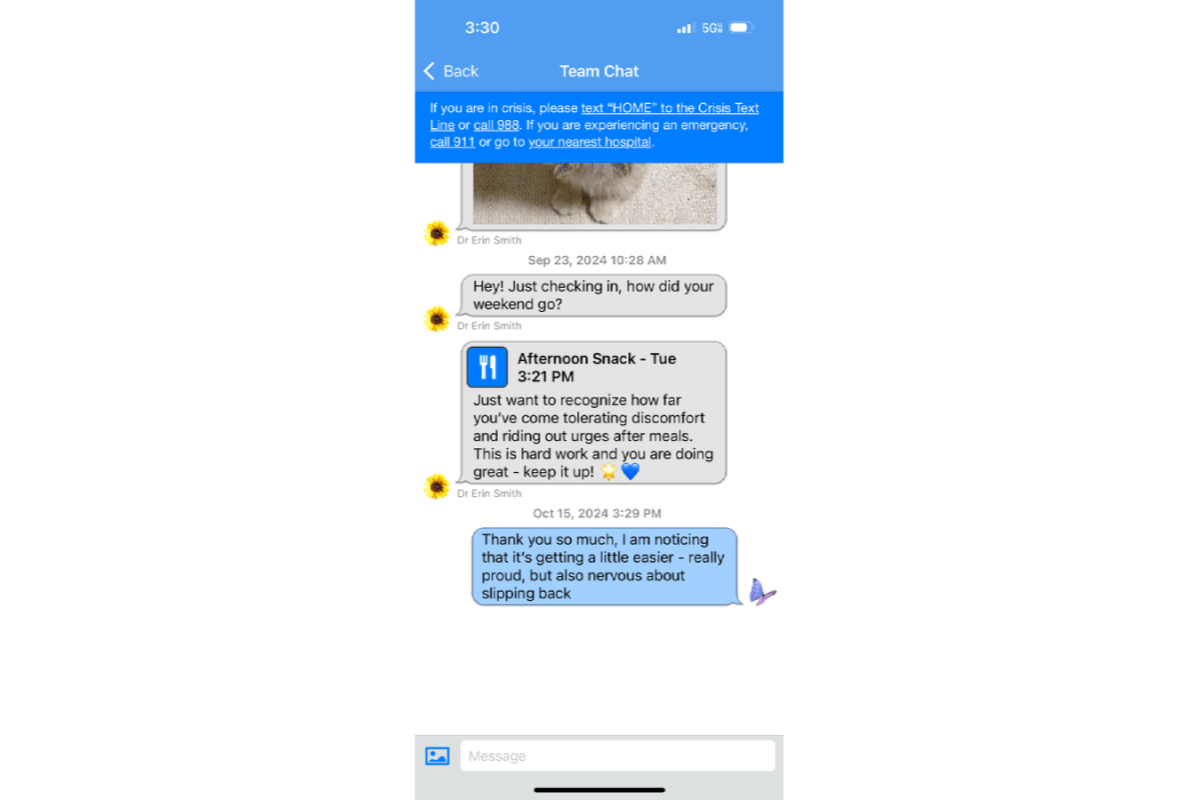
Messaging feature of the Recovery Record app.

**Figure 4:**
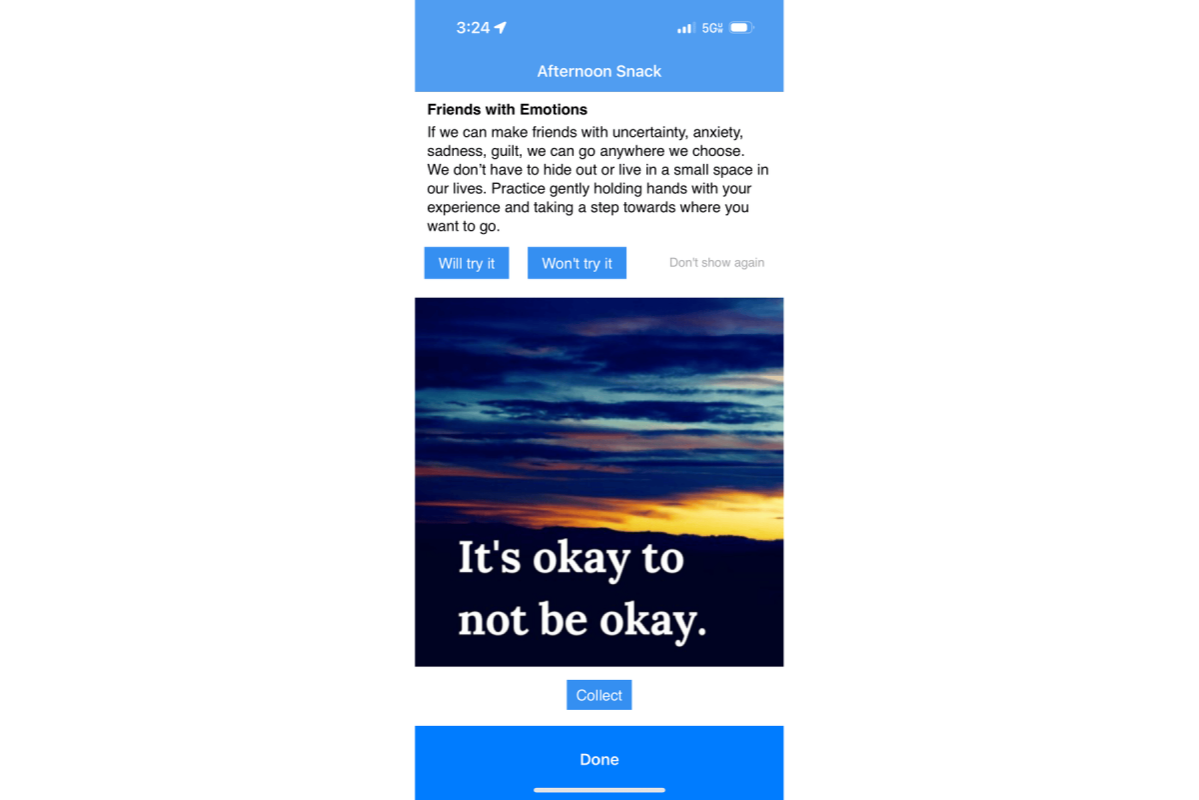
Coping skills and affirmations within the Recovery Record app.

In addition to daily self-monitoring tasks, RR users received a program of core cognitive-behavioral techniques, such as cognitive restructuring (ie, instead of telling yourself “I am fat and disgusting,” change it to: “my eating disorder is telling me I am fat and disgusting”), stimulus control (ie, reduce the chance of binge eating by taking only enough cash with you to pay for necessities), and delaying tactics (ie, putting off acting on an urge for a specific amount of time), along with therapeutic goals, which were reviewed and personalized by their therapist, on a weekly basis. Content was tailored in accordance with the users’ diagnosis, which was provided by their therapist, and the contents of their CBT meal monitoring entries. A detailed description of the RR patient app has been published previously [11], and screenshots of the app are shown in Figures 1-4.

### Outcomes Measures

These comprised 2 large groups: health care service utilization and health outcomes. Health care service utilization measures included encounters relevant to outpatient treatment compliance and retention, namely, outpatient psychiatric visits completed. In addition, we included emergency department visits, with associated *ICD-10* ED diagnosis, as a health service utilization measure that may be indicative of escalation of ED acuity. Given that ED often requires medical management, records of outpatient visits with primary care physician including an associated ED diagnosis were also obtained. Health outcomes included changes in BMI, changes in blood pressure (BP), and depressive symptoms, which were measured using the 9-item Patient Health Questionnaire (PHQ-9) [24].

### Statistical Analysis

A summary of the baseline characteristics of the study population was conducted, using frequencies and means as appropriate. We tested for differences between AAT and standard of care using chi-square tests for categorical variables and 2-sample *t* tests for continuous variables.

The primary outcomes included health care service utilization (ie, the number of outpatient psychiatric visits, outpatient primary care visits, and emergency department visits). Secondary outcomes included health outcomes: BMI change and BP (systolic and diastolic) change. An exploratory outcome included depression scores as measured by the PHQ-9.

To address confounding by observed covariates and reduce the selection bias that exists in the absence of randomization, we used inverse probability of treatment weighting (IPW) for estimating causal effects. IPW is a type of weighting method whose goal is to make the observed sample as representative of the population by weighting outcomes according to the inverse of the probability of treatment assignment [25]. Weights are obtained from a treatment selection model, estimated using logistic regression with the treatment assignment as the dependent variables and the baseline characteristics as independent variables, that is, gender, age at ED diagnoses, current age, treatment facility, history of depression, history of anxiety, history of substance abuse, BP, BMI, history of any intensive outpatient admission, any intensive outpatient admission during the study period, and diagnosis type.

The average treatment effect (ATE) was defined as the mean difference between AAT and TAU with respect to number of outpatient and emergency department visits (primary outcome). For the secondary outcome, ATE was defined by the difference in weighted averages of BMI and BP obtained from the treated and TAU groups. For the exploratory outcome of depression scores, the ATE was defined as the mean difference between AAT and TAU with respect to change in PHQ category.

Significance tests and CIs were based on SEs obtained from the bootstrap method (5000 replications) to calculate the variance of the weighted average of outcomes. We used an a value of .05 for significance, and all tests were 2-sided. A complete case analysis was performed on the primary outcomes, meaning that patients who were missing utilization outcomes data were not included in the primary analysis.

The final analytic cohort included 1154 patients who had complete data on pre- and postintervention BMI as well as all demographic variables needed for inclusion in the weighting model. For the analysis on health care utilization, 1104 (AAT: n=165, TAU: n=939) individuals had complete information on outpatient primary care visits, 939 (AAT: n=173, TAU: n=766) individuals had completed outpatient psychiatric visits, and 222 individuals had complete information on emergency department visits. The analysis on health outcomes excluded individuals who had vitals taken less than 30 days apart, resulting in 535 individuals. The exploratory analysis on depression scores excluded individuals who had taken the PHQ-9 fewer than 30 days apart, resulting in 441 individuals. All analyses were performed using R Statistical Software (version 4.1.2; R Core Team 2021).

## Results

### Patient Characteristics

Baseline characteristics of patient population are shown in Table 2. Patients in the AAT group were younger, with a mean age of 29 (SD 14) years, than those in the TAU group, with a mean age of 38 (SD 18) years (*P*<.001); they also had a lower mean BMI of 24.1 (SD 8.4) kg/m^2^ compared with 26.8 (SD 9.4) kg/m^2^ (*P*<.001). More patients in the AAT group were categorized as underweight (*P*<.001) and having a history of hypotension (*P*=.004), with a lower mean resting systolic BP (*P*=.003). In addition, more patients in the AAT group had a history of a depressive disorder diagnosis (*P*=.05) and anxiety diagnosis (*P*<.001); engagement with Intensive Outpatient Programs (IOPs; *P*<.001); and a current diagnosis of AN (*P*<.001). Conversely, there were more patients in the TAU group with a diagnosis of BN (*P*<.001) and other EDs (*P*<.001).

**Table 2:** Patient characteristics.

Variable			AAT^a^ (n=174)		TAU^b^ (n=971)		*P* value
**Age (year), mean (SD)**			29 (14)		38 (18)		<.001
**Sex, n (%)**							.20
	Female	163 (93.7)		878 (90.4)			
	Nonfemale	11 (6.3)		93 (9.6)			
**Resting blood pressure (mm Hg), mean (SD)**							
	Systolic	113 (15)		116 (15)		.003	
	Diastolic	68 (10)		69 (10)		.05	
**Blood pressure category^c^, n (%)**							.004
	Hypotensive	29 (17)		145 (15)			
	Normal	99 (57)		424 (44)			
	Elevated	27 (16)		225 (23)			
	High	19 (10)		177 (18)			
**BMI (kg/m^2^), mean (SD)**			24.1 (8.4)		26.8 (9.4)		<.001
**BMI category^d^, n (%)**							<.001
	Underweight	43 (24.6)		126 (12.9)			
	Normal	81 (46.6)		426 (43.9)			
	Overweight	20 (11.5)		152 (15.7)			
	Obese	13 (7.5)		92 (9.5)			
	Severe obesity	17 (9.8)		175 (18)			
**Eating disorder diagnosis, n (%)**							<.001
	Anorexia nervosa	61 (35.1)		171 (17.6)			
	Bulimia nervosa	23 (13.2)		219 (22.6)			
	Eating disorder not otherwise specified	66 (37.9)		368 (37.9)			
	Other eating disorder	24 (13.8)		213 (21.9)			
**History of depressive disorder, n (%)**			123 (70.7)		608 (62.6)		.05
**History of anxiety disorder, n (%)**			138 (79.3)		612 (63)		<.001
**History of intensive outpatient program treatment, n (%)**			90 (51.7)		76 (7.8)		<.001

^a^AAT: app-augmented treatment.

^b^TAU: treatment as usual.

^c^Blood pressure categories were defined as hypotension: <90 mm Hg systolic or <60 mm Hg diastolic, normal: 90-120 mm Hg systolic or 60-80 mm Hg diastolic, elevated: 120-139 mm Hg systolic or 80-89 mm Hg diastolic, and high: ≥140 mm Hg systolic or ≥90 mm Hg diastolic.

^d^BMI categories defined as underweight: <18.5 kg/m^2^, normal: ≥18.5 to <25 kg/m^2^, overweight: ≥25 to <30 kg/m^2^, and obese: ≥30 to <40 kg/m^2^.

### Changes in Patient Health Care Utilization

Patients in the AAT group had an average of 28.7 outpatient psychiatric visits compared with patients in the TAU group, who had an average of 18.5 outpatient psychiatric visits, which was a significant difference (treatment effect [TE] 10.2, 95% CI 10.0-10.41; *P*<.001). Patients in the AAT group had fewer emergency department visits (mean 0.14) than patients in the TAU group (mean 0.50; difference of 0.36, 95% CI –0.55 to –0.18; *P*<.001; Table 3). There was no significant difference in primary care outpatient utilization, with an average of 4.7 visits in the AAT group and 5.1 visits in the TAU group (*P*=.30).

**Table 3:** Weighted means of patient health care utilization.

	AAT^a^, mean	TAU^b^, mean	Difference (95% CI)	*P* value
Emergency department visits (n=222)	0.14	0.50	–0.36 (–0.55 to –0.18)	<.001
Outpatient psychiatry visits (n=939)	28.69	18.49	10.2 (10.0 to 10.41)	<.001
Outpatient primary care visits (n=1104)	4.82	5.10	–0.28 (–1.46 to 0.89)	.30

^a^AAT: app-augmented treatment.

^b^TAU: treatment as usual.

### Changes in Patient ED Outcomes

Patients in the AAT group experienced a larger increase in BMI after treatment than those in the control group (0.75 compared with 0.31; TE 0.44, 95% CI –0.05 to 0.92; *P*=.02; Table 4). Furthermore, a significantly higher proportion of patients in the AAT group changed into a lower BMI category compared with patients in the TAU group (TE 0.19, 95% CI 0.05-0.33; *P*=.01). Notably, patients in the AAT group who were underweight at baseline had nearly a 1 weight category increase (0.89 compared with 0.24 in the TAU group; TE 0.65, 95% CI 0.35-0.95; *P*<.01). There was a significant difference in resting systolic BP, with the AAT group experiencing a larger change (TE –2.30, 95% CI –2.38 to –2.22; *P*=.05), although there was no significant difference in diastolic BP (TE –1.29, 95% CI –6.38 to 3.79; *P*=.11).

**Table 4:** Weighted mean differences in patient treatment outcomes.

	AAT^a^ (n=174), mean difference	TAU^b^ (n=971), mean difference	Treatment effect difference (95% CI)	*P* value difference
BMI	0.75	0.31	0.44 (–0.05 to 0.92)	.02
BMI category	0.30	0.11	0.19 (0.05 to 0.33)	.01
Systolic blood pressure	–2.08	0.22	–2.30 (–2.38 to –2.22)	.05
Diastolic blood pressure	–1.55	–0.3	–1.29 (–6.39 to 3.79)	.11
Blood pressure category	–0.15	–0.057	–0.093 (–0.28 to 0.10)	.20
Depressive symptoms (n=441)	–2.44	–1.48	–0.96 (0.027 to –1.94)	.03

^a^AAT: app-augmented treatment.

^b^TAU: treatment as usual.

### Changes in Patient Depressive Symptoms

Patients in both the AAT group and the TAU group showed significant improvement in depressive symptoms at the end of treatment as measured by the change in raw PHQ-9 scores (pre-post difference of –2.44, 95% CI –3.39 to –1.48 and –1.5, 95% CI –1.72 to –1.24, respectively; *P*<.001 for both). Moreover, patients in the AAT group showed significantly greater improvement in depressive symptoms than patients engaging in the TAU group (TE –0.96, 95% CI 0.027 to –1.94; *P*=.03). This was a clinically significant change, with those in the AAT group showing a larger PHQ-9 change in terms of units of category changes compared with TAU patients (difference –0.22, 95% CI –0.417 to 0.015; *P*=.015).

## Discussion

### Principal Findings

Our research addresses an important evidence gap regarding the potential of digital therapeutics for EDs to impact health service utilization and health outcomes when implemented in a real-world health system. The results of this study suggest that AAT may increase participation in outpatient ED treatment and result in a significant reduction in disorder-related emergency department visits, which are a large component of associated disease-specific costs [9]. Furthermore, in this study, AAT resulted in significantly and clinically improved health outcomes, with faster weight stabilization in low-weight patients and a greater reduction in depression symptoms.

RR includes several capabilities that automated best practice components of treatment. Patients using the RR mobile app are prompted to complete meal-monitoring entries throughout the day and are systematically introduced to tailored therapeutic coping strategies and clinical goals. The self-monitoring component of the app is provided in an evidence-based CBT format. Meal monitoring is a fundamental component of CBT for EDs and comprises a standard CBT-style question set (what was eaten, with whom, was it sufficient, etc) and some optional additional EDs symptom-focused questions such as urges to engage in compensatory behaviors, current emotional state, compulsive exercise, sleeping patterns, hunger levels, intrusive thoughts, and coping skills used (if any). These questions serve to promote awareness of the nature of the eating problem and identification of contextual and personal triggers that are maintaining disordered behaviors, in addition to the promoters of and barriers to change. Mealtime self-monitoring also creates a structure for routinized eating, which has been found to disrupt dietary restriction and binge eating patterns [26]. In a prior study that included 458 individuals who used the RR app for self-monitoring without any additional support, 35% of users were classified as in remission and 46% achieved a clinically meaningful reduction in ED symptoms, as per the Eating Disorder Examination Questionnaire, after 1 month [27]. Furthermore, a qualitative study on use of the RR app describes how patients reported that it helped them become more aware of their eating habits and feel more in control of their recovery [17].

A major strength of this study is its examination of the real-world effects of the implementation of an ED app on health service utilization and clinical outcomes. This analytic approach was made possible due to a multiorganization collaboration that included academics, researchers at a DTx organization, and clinical researchers from a large HMO. Collaboration with the integrated HMO and integration of the RR app with therapist care created the unique opportunity to access a complete medical record data set on clinical and health care service utilization outcomes, allowing for a retrospective analysis in which a causal TE could be estimated.

Moreover, this study took place within a system that treats racially and diagnostically diverse patients and in which therapists are often tasked with managing large caseloads with constrained resources, while faced with high dropout from outpatient services and a high volume of emergency admissions for ED. Given that the most recent metareviews of the literature on treatment of ED have called out an “urgent need for novel treatments, particularly in AN [28],” the findings from this study shed light on the real-world potential of implementing an innovative digital solution in outpatient and intensive outpatient services, with the ultimate goal of helping patients stick to their evidence-based treatment plans for long enough to achieve clinical outcomes.

Due to the fact that this study was conducted in an observational setting, some baseline differences are due to implementation factors within the HMO. For example, the app was implemented extensively in the IOP during the study period, which explains why the history of IOP is higher among the AAT group. Related, younger patients, as well as patients with AN, were disproportionately represented in the AAT group. It is possible that this difference may be attributed to the greater level of severity or medical complexity typically associated with AN and higher levels of participation of younger patients with AN in intensive treatment programs. Given that an RCT was not feasible in this real-world setting, we used a robust statistical method of IPW to adjust for such baseline between group differences. We explored alternative methodologies such as covariate balancing via propensity score matching, where a treatment subject would be matched to a set of control participants; however, we found that this approach discarded many control participants and thus we ultimately selected a method that would permit use of all available data with the most robust analysis.

While our study provides insight into the potential for digital recovery solutions to be integrated into ED treatment within a real-world health care system, there are limitations to this approach that are worth noting. For example, at the time of the study, KPNC used various partners for residential and partial hospitalization programs with outside vendors that could not be extracted for inclusion in this study. In the absence of available data, we were unable to evaluate the potential impact of AAT on admissions or readmissions to these high-acuity and often high-cost programs. Another limitation of this retrospective research design is the absence of a standardized psychometric measure of ED pathology in the TAU group. Without these data for comparison, we were unable to fully explore the potential impact of AAT on all clinical outcomes of interest. Finally, the study was conducted among a stably insured population within an integrated health care delivery system with a focus on preventive care. In this treatment context, both therapists and patients may have been more likely to have adequate resource to participate in app-augmented therapist–guided care. Thus, the findings may not generalize to the overall US population without similar levels of insurance and access to care.

Future work deriving from this study could include a more in-depth exploration of the demographic and clinical characteristics that influence engagement with and response to the app. Researching the differences in the effectiveness of the app-enhanced program among different age groups, gender, and severity of ED could provide insightful information to clinicians as they consider how to further maximize the benefits of incorporating the technology in their clinical practice. Given the behavioral health outcome findings, further investigation into the AAT’s potential in addressing comorbid mental health conditions associated with EDs such as depression would be of added value. Furthermore, studying the long-term costs and clinical effectiveness would be necessary to gain insights into the effects of the app on maintaining improvement in health outcomes and preventing relapses and readmissions at a sustained cost reduction via lower health care utilization. Finally, it is important to note that not everyone engages with the app at a similar rate, and understanding usage rates and engagement with the different tools and features of the app can help identify to what degree is engagement itself positively associated with health care utilization and clinical outcomes, and how much usage may be needed to gain the benefits suggested by this study.

### Conclusions

This study provides evidence that integrating the use of a remote patient engagement app into the treatment plan for patients with ED has beneficial effects. Particularly, AAT led to significant improvements in BMI for underweight patients and in depressive symptoms when compared with conventional treatment methods. The AAT was also associated with fewer emergency department visits and increased participation in outpatient treatment programs. This suggests that digital interventions could play a key role in managing these health conditions and potentially reducing health care service utilization and associated costs. This study serves as a promising point of reference for future research on the long-term efficacy, cost-effectiveness, and potential impact of leveraging digital health interventions to better treat EDs.

## Abbreviations

AAT: app-augmented treatment
AN: anorexia nervosa
ATE: average treatment effect
BED: binge eating disorder
BN: bulimia nervosa
BP: blood pressure
CBT: cognitive behavioral therapy
DTx: digital therapeutic
ED: eating disorder
HMO: health maintenance organization
ICD-10: International Classification of Diseases, Tenth Revision
IOP: Intensive Outpatient Program
IPW: Inverse probability of treatment weighing
KPNC: Kaiser Permanente Northern California
PHQ-9: 9-item Patient Health Questionnaire
RCT: randomized controlled trial
RR: Recovery Record
TAU: treatment as usual
TE: treatment effect
